# Data on overstory and understory trees in aspen-dominated boreal mixedwood stands over 20 years after partial harvesting

**DOI:** 10.1016/j.dib.2018.01.019

**Published:** 2018-01-20

**Authors:** Rongzhou Man, Hua Yang, John W. Schnare

**Affiliations:** a Ontario Forest Research Institute, Ontario Ministry of Natural Resources and Forestry; b College of Forestry, Beijing Forestry University

## Abstract

Growing demand for non-timber forest ecosystem services has resulted in increased use of partial harvesting in boreal forests. Since the 1990s, multiple studies have yielded short-term responses to partial harvesting. Here we present an inventory of longer-term (20 years) responses of overstory and understory trees to partial harvesting in aspen-dominated boreal mixedwood stands. Pre- and post-harvesting overstory trees were mapped and measured for total height and diameter at breast height (DBH); understory trees were measured for total height. Codes identify tree species, treatments, and years since harvest. Data are stored in separate Microsoft Excel spreadsheets: overstory trees, understory trees, and years after harvesting.

**Specifications Table**

**Table:** 

Subject area	*Forest ecology*
More specific subject area	*Tree inventory data*
Type of data	*Table*
How data was acquired	*Field measurements*
Data format	*Raw, filtered*
Experimental factors	*Harvesting, forest tent caterpillar defoliation*
Experimental features	*Unharvested, partially harvested, and clear cut*
Data source location	*40 km east of Cochrane, northeastern Ontario, Canada*
Data accessibility	*With this article*
Related research article	*Yang, H., and Man, R. Effects of partial harvesting on species and structural diversity in aspen-dominated boreal mixedwood stands. For. Ecol. Manage. (2018) 409:653–659*.

**Value of the data**

• Overstory and understory tree data assessed at 0, 1, 3, 5, 11, and 20 years post-harvesting.
• Potential to analyze long-term dynamics of boreal forests following partial harvesting and forest tent caterpillar outbreaks.
• Opportunity to link short and long-term responses and assist resource managers in projecting long-term stand density, composition, and yield.
• Opportunity to study stand-scale mortality and ingrowth processes using mapped tree locations.

## 1 Data

The overstory and understory tree data presented here were the basis for the research article by Yang and Man [1] and the method documented by Man and Yang [2]. The raw data analyzed by Yang and Man [1] are available as Microsoft Excel spreadsheets in the Supplementary Material. An excerpt of the overstory data (Partial cut_Overstory) is shown in Table 1 and the understory data (Partial cut_Understory) in Table 2. Note: Each year's data is stored in a separate worksheet. Previously reported post-harvesting data includes 5- and 11-year regeneration analyses [3], [4] and 11-year responses of overstory trembling aspen to harvesting and forest tent caterpillar defoliation that occurred 3 to 5 years after harvesting [5].

**Table 1:** Excerpt of overstory data provided in the Supplementary Material.

Overstory plot #	Section	Tree #	Species code	Distance (m)	Azimuth (°)	DBH (cm)	Height (m)	Survival code
1	1	1	2	6.70	16.7	16.7	17.70	2
1	1	2	4	8.80	16.7	20.8	22.70	1
1	1	3	3	10.40	22.5	17.1	11.70	1
1	1	4	4	10.70	19.0	27.4	23.30	1
1	1	5	11	12.10	25.6	28.7	27.60	1
1	1	6	11	14.80	4.6	31.4	24.90	1
1	2	1	2	5.60	37.6	23.0	17.90	1

**Table 2:** Excerpt of understory data provided in the Supplementary Material.

Overstory plot #	Understory plot #	Species code	Height (cm)
1	5	1	114.0
1	10	3	47.0
1	11	2	40.0
2	4	1	60.0
3	2	1	76.0
3	3	1	3.0
7	1	1	1.5

## 2 Experimental design, materials and methods

Data presented here is from a partial harvesting experiment established in the early 1990s. The initial design was a randomized complete block with 4 harvesting treatments replicated 4 times. The study was originally designed to remove 0 (unharvested), 36 and 68% (partially harvested), and 100% (clearcut) of the merchantable overstory basal area (BA) of all trees ≥10 cm DBH. During application, however, the design was adjusted with the actual basal area removal approaching 40% for the partial harvesting treatments. Adjustments were necessary because of limiting operational site characteristics and the overarching need to protect advance conifer regeneration, resulting in 8 replications of 40% partial harvesting [3], [6]. During January–February 1995, the 100 m×100 m harvesting plots were full-tree logged with a feller-buncher and a grapple skidder. To achieve the 40% harvesting level, 20% of the overstory was removed, focusing on large trembling aspen and balsam fir in 16-m-wide leave strips plus 5-m-wide skid trails that were clear cut. To minimize machinery damage to residual trees, harvesting was done using careful logging techniques [6].

After harvesting, overstory plots, with a 25 m radius (0.2 ha) divided into 12 sections of 30 degree segments, were established in the centre of the 1-ha harvested area. Pre- and post-harvesting measurements included DBH and height for all tree species ≥4.0 m, survival status (live=1 and dead=2), as well as the location, distance and azimuth, of individual stems in relation to plot centre. On the circumference of the overstory plots, 12 equally spaced 2 m×2 m understory plots were established. Within the understory plots, all tree species <4.0 m were identified and measured for total height.
